# The triglyceride glucose-body mass index is positively associated with higher risk of hypertension in rural southwest Chinese population: a cross sectional study

**DOI:** 10.3389/fcvm.2025.1677048

**Published:** 2026-02-02

**Authors:** Mingxue Dong, Ming Ma, LiJing Yang, Huali Xiong

**Affiliations:** 1 The First People’s Hospital of Shuangliu District, Chengdu, Sichuan Province, China; 2 Center for Disease Control and Prevention of Rongchang District, Chongqing, China; 3 Health Committee of Rongchang District, Chongqing, China

**Keywords:** body mass index, glucose, hypertension, insulin resistance, triglyceride, TyG-BMI

## Abstract

**Background:**

Insulin resistance (IR) has been shown to be associated with hypertension. The triglyceride-glucose body mass index (TyG-BMI) has emerged as a novel surrogate marker for assessing IR. This study aimed to investigate the association between the TyG-BMI index and hypertension among adults in rural southwest China using a cross-sectional study.

**Method:**

We recruited 2,998 people between the ages of 30 and 79 from Rongchang, Chongqing municipality, southwest China, as part of The China Multi-Ethnic Cohort Study, considered the largest cohort study in southwest China. Logistic regression model, restricted cubic spline (RCS) model and receiver operating characteristic (ROC) were applied to estimate the association between the TyG-BMI index and hypertension. Moreover, subgroup and sensitivity analyses were undertaken to check the consistency of the outcomes.

**Results:**

A total of 2,998 participants were included in the present study, with a hypertension prevalence of 39.93%. After adjusting for confounding factors, the ORs of hypertension in Q2, Q3, Q4 were 1.641 (1.277–2.109), 1.768 (1.371–2.281), 2.463 (1.794–3.382) compared with the lowest quartile (Q1), respectively. RCS indicated that the TyG-BMI index was nonlinearly associated with hypertension (*P*_for overall_ < 0.001, *P*_for nonlinea*r*_ = 0.046). The ROC analysis indicated that the TyG-BMI index had a 64.1% (AUC: 0.641, 95% CI: 0.621–0.661) ability to distinguish hypertension. Subgroup analysis in participants without diabetes, dyslipidemia, hyperuricemia and central obesity as well as sensitivity analyses also demonstrated the similar relationship between the TyG-BMI index and hypertension.

**Conclusion:**

The current study demonstrates that the TyG-BMI index is associated with higher risk of hypertension among rural adults in southwest China. Lifestyle modifications, including weight control, vigorous physical activity and healthy dietary pattern can help improve IR and prevent hypertension.

## Introduction

Hypertension has been identified as a major public health challenge in low- and middle-income countries (1). In 2021, hypertension accounted for the highest burden among the five most common metabolic diseases globally, with 226 million disability-adjusted life years (2). The number of adults with hypertension is projected to increase by approximately 60% by 2025 compared to 2000, reaching an estimated 1.56 billion cases, with a significant concentration in economically developing countries (3). According to the Report on Cardiovascular Health and Diseases in China (2023 edition), the prevalence of hypertension among Chinese adults is 31.60% (4), while the rate of awareness, treatment, and control of hypertension was relatively low in China (5). Hypertension accounted for 6.60% of total health expenditures in China, imposing a substantial disease burden (6). Given its high prevalence and significant economic impact, the use of a simple and rapid indicator for early detection would be highly valuable.

Insulin resistance (IR) is a major contributor to cardiovascular diseases, hypertension, and metabolic disorders (7, 8). Early detection of IR is crucial for effective disease prevention and control. A meta-analysis demonstrated that IR was significantly associated with an increased risk of developing hypertension (9). The primary mechanisms by which IR elevated blood pressure included increased tissue activity of angiotensin II and aldosterone (10, 11), oxidative stress and the endothelial insulin resistance phenomenon (12). Hyperinsulinemic-euglycemic clamp (HIEC) (13) and homestasis model assessment for insulin resistance (HOMA-IR) (14) are currently used for the diagnosis of IR. However, due to their high cost, complex procedures, and poor reproducibility, these two diagnostic methods are not widely used in clinical practice (15). In recent years, the triglyceride-glucose (TyG) index and the triglyceride-glucose body mass index (TyG-BMI) have emerged as alternative markers for assessing IR. These indices can be calculated using fasting triglycerides (TG), fasting blood glucose (FBG), and body mass index (BMI). A recent study (16) has demonstrated that the TyG-BMI index is a more effective surrogate marker for IR. It has emerged as a novel, cost-effective, and efficient tool for evaluating IR in sudden cardiac arrest (17), pre-hypertension (18), pre-diabetes (19), hyperuricemia and non-alcoholic fatty liver disease (20), which has gained increasing recognition and growing interest in this field.

Previous studies have investigated the association between the TyG-BMI index and hypertension (21–25). However, their findings have been inconsistent, largely due to small sample sizes or a lack of representative populations. Moreover, data on the relationship between the TyG-BMI index and hypertension in the general population of rural southwest China remain limited. Therefore, the present study aimed to examine the association and dose-response relationship between the TyG-BMI index and hypertension in this region.

## Method

### Study design

The participants were from the baseline cross sectional survey of the China Multi-Ethnic Cohort Study (26), representing the most extensive cohort investigation carried out by Sichuan University in Southwestern China between September 2018 and January 2019. Specifically, in the Rongchang area (27), participants were recruited using a three-stage stratified random sampling method. First, four streets, Changyuan, Changzhou, Anfu, and Guangshun were randomly selected from a total of 21 streets or towns. Subsequently, 10 villages were randomly chosen from each selected street. Finally, 50–80 individuals were randomly selected from each village, ensuring that the age and sex distribution was representative of the Rongchang population. Ethical approval was obtained from the Ethics Committee of Sichuan University (No. K2016038), and all participants provided written informed consent prior to participation in the survey.

### Study population

Participants in this study were selected according to the following inclusion criteria: (1) age ranging from 30 to 79 years during 2018; (2) residence in Rongchang for at least six months; (3) Han ethnicity; (4) voluntary engagement in the study, signing informed consent, and agreeing to donate biological samples and participate in follow-up evaluations; (5) absence of mental illness, cognitive impairment, or communication difficulties. Individuals were excluded if they had incomplete data on basic demographics, questionnaires, physical examinations, blood biochemical analyses. A total of 3,002 participants were initially recruited in the baseline survey. However, those with missing data were excluded. Finally, 2,998 participants were included in the analyses to examine the relationship between the TyG-BMI index and hypertension.

### Data collection

Before the initiation of the survey, all interviewers, physicians, and investigators received comprehensive training and were evaluated by the quality control team. The training covered the use of tablets or computers to administer questionnaires, verification and uploading of completed questionnaires, standardized procedures for physical examinations, and protocols for sample collection and transportation. Verified questionnaires were uploaded after reviewed by designated staff. Trained postgraduate students from Sichuan University reviewed 1% of the questionnaires daily. Any issues identified were promptly reported to the investigators for follow-up. All blood sample analyses were conducted by Chongqing Di'an Medical Testing Center Co., Ltd.

### Assessment of covariates

Well-trained interviewers collected information on demographic and lifestyle factors, including age, gender, marriage status, education level, job, and total family income, as well as smoking and drinking status, physical activity (PA) levels, dietary approaches to stop hypertension (Dash) scores, and night sleep duration. Physical examination data such as systolic and diastolic blood pressure (SBP/DBP), height, body weight, waist circumference (WC) were also obtained. Additionally, venous blood samples were drawn to assess fasting blood glucose (FBG), total cholesterol (TG), triglyceride (TC), high-density lipoprotein cholesterol (HDL-C), low-density lipoprotein cholesterol (LDL-C), uric acid (UA).

Gender was classified into two groups: males and females. Marriage status was divided into two categories: married/cohabiting, separated/divorced/widowed and unmarried. Educational level was grouped into three levels: primary school or below, junior middle school, and high school or above. Job was categorized into five groups: farmers, government employees, workers, sales staff and others. Total family income was stratified into four brackets: less than 20,000 yuan, 20,000–59,999 yuan, 60,000–99,999 yuan, and 100,000 yuan or more. Smoking status was classified as No or Yes based on the total consumption of cigarettes over 100 lifetime. Drinking status was also classified as as No or Yes based on the question “whether you have drinking alcohol last 30days before the survey”. PA levels were assessed by metabolic equivalent tasks (METs) (28), which included the extent of physical activity in work, transportation, household responsibilities, and leisure activities. Total weekly activity level less than 600 (MET-min/w) was defined as low, total weekly activity level between 600 and 3000 (MET-min/w) was defined as moderate and total weekly activity level over 3,000 (MET-min/w) was defined as vigorous (29). A modified version of the DASH score was obtained using the procedure established by Chiu and Chen (30, 31), with minor adjustments based on the CEMC data (32). This modified DASH score emphasized 7 main food: whole grains, fresh fruits, fresh vegetables, legumes, dairy products, red meat items, and sodium intake. Scores ranging from 1 to 5 were allocated according to the quintiles of average consumption for each food group. Specifically, a score of 5 was assigned to the highest quintile of intake for whole grains, fresh fruits, fresh vegetables, legumes, and dairy products, while the lowest quintile received a score of 5 for red meat items and sodium intake. The overall DASH score for each participant was then derived by summing the scores of these seven food groups (33). Using the lowest tertile of the DASH score as a threshold, participants were classified into two groups: “<20” and “≥20”. Night sleep duration was defined according to the sleep guidelines of national lung and blood institute, the recommended daily sleep duration for adults is 7–8 h per day. Sleep duration <7 h/day is defined as insufficient sleep, 7–8.9 h/day as adequate sleep, and ≥9 h as excessive sleep (34). Hypertension (35) was identified as: (1) having an average SBP of ≥140 mmHg or an average DBP of ≥90 mmHg, based on three consecutive measurements taken at 5 min intervals while the individual was at rest or (2) self-reported previously having been diagnosed with hypertension by a physician or (3) engaging in efforts to reduce blood pressure (e.g., medications, lifestyle modifications). Diabetes (36) was identified in individuals with FPG levels exceeding 7.00 mmol/L, or in those who previously had been diagnosed with diabetes by a physician or were actively managing their FPG levels through interventions such as medications or lifestyle changes. Dyslipidemia (37) was diagnosed in the presence of any of the following four lipid abnormalities: (1) TC ≥ 6.2 mmol/L; (2) TG ≥ 2.3 mmol/L; (3) LDL-C ≥ 4.1 mmol/L; (4) HDL-C < 1.0 mmol/L. Overweight and obesity was defined by body mass index (BMI). BMI was computed by dividing body weight by the square of height (kg/m^2^). Participants with a BMI of 24–27.9 kg/m^2^ were classified as overweight, while those with a BMI of ≥28 kg/m^2^ were categorized as obesity according to The Expert Consensus on Obesity Prevention and Treatment in China (38). Hyperuricemia was defined by UA. Participants with a UA over 420 μmol/L were classified as hyperuricemia (39). Central obesity was defined by WC. Males with a WC over 90 cm and females with WC over 85 cm were classified as central obesity (40).

### The definition of TyG-BMI index

TyG-BMI index was calculated by the following formulas (21):

$$\mathrm{TyG}\text{-}\mathrm{BMI}\;\mathrm{index}=Ln\left[\frac{\mathrm{TG}(\mathrm{mg}/\mathrm{dL})\times \mathrm{FBG}(\mathrm{mg}/\mathrm{dL})}{2}\right]\times \mathrm{BMI}$$

The TyG-BMI index was divided into four groups based on quartiles: Q1 (<20.69), Q2 (20.70–31.39), Q3 (31.40–44.84), and Q4 (>44.85).

### Statistical analyses

Baseline characteristics of the study participants were presented by the presence or absence of hypertension and quartiles of the TyG-BMI index (Q1, Q2, Q3, Q4). Continues variables with normal distribution were presented as mean ± standard deviation. Continues variables with non-normal distribution were presented as medium and inter quartile range. Categorical variables were presented as number and percentage (%). Independent-sample *t* test, Mann–Whitney *U* test, Kruskal Wallis *H* test, or chi-square test were applied as appropriate. Odds ratio (OR) with 95% confident interval (CI) were calculated to explore the association between the TyG-BMI index (Q1, Q2, Q3, Q4) and hypertension by logistic regression analyses. The first quartile (Q1) was served as the reference, while the 2nd to 4th quartiles (Q2, Q3, Q4) were compared with Q1. Multicollinearity among the logistic regression variables was assessed using variance inflation factor (VIF) diagnostics, with a VIF of less than 5 indicating its absence. In crude model, no factors were adjusted. In adjusted model I, age, gender, marriage status, education level, job, total family income were adjusted in the logistic model. In adjusted model II, age, gender, marriage status, education level, job, total family income, smoking status, drinking status, PA level, DASH score, night sleep duration, diabetes, dyslipidemia, hyperuricemia, central obesity were adjusted in the logistic model. Next, To further explore the potential complex nonlinear associations and visualize dose-response relationship between TyG-BMI index and hypertension, the crude and multivariate- adjusted restricted cubic spline (RCS) were applied. To ensure the robustness of the study, a sensitivity analysis was performed by excluding participants with diabetes, dyslipidemia, hyperuricemia and central obesity to evaluate the robustness of the primary findings by logistic regression (adjusted model III) and RCS. In adjusted model III, age, gender, marriage status, education level, job, total family income, smoking status, drinking status, PA level, DASH score, night sleep duration were adjusted in the logistic model. Receiver operating characteristic (ROC) analyses was generated to assess the predictive capacity of the TyG-BMI index for hypertension, utilizing the area under the curve (AUC) and its corresponding 95% confidence interval. The analysis was conducted by R software (R4.3.1) and *p* value <0.05 with two side was considered statistically significant.

## Result

### Baseline characteristics of study participants with and without hypertension

A total of 2,998 participants were included in the current study with a medium age of (49, *P*_25_ ∼ *P*_75_: 42, 60) years, of which 1498 were males, accounting for 49.97%. The prevalence of hypertension in study participants was 39.93% (1,197/2,998). The baseline characteristics of study participants with and without hypertension were presented in Table 1. Participants with hypertension exhibited significantly higher levels of age, TC, LDL-C, TG, FBG, BMI, WC, UA, SBP, and DBP compared to non-hypertensive participants. However, participants without hypertension had a significantly higher level of HDL-C compared to hypertensive participants (*P* < 0.001). The medium index of TyG-BMI in hypertensive participants was 36.91, which was significantly higher than that in participants without hypertension (28.08) (*P* < 0.001). The significant differences in proportion of gender, marriage status, education level, job, total family income, PA level, Dash score, night sleep duration, diabetes, dyslipidemia, hyperuricemia, overweight/obesity, central obesity were found (*P* < 0.05) (Table 1).

**Table 1:** Baseline characteristics of study participants with and without hypertension.

Variables	Total (*n* = 2,998)	Hypertension		Statistic value	*P* value
		No (*n* = 1,801)	Yes(*n* = 1,197)		
Age, M (Q_1_, Q_3_)	49.00 (42.00, 60.00)	45.00 (38.00, 52.00)	56.00 (48.00, 66.00)	*Z* = −24.373	<0.001
TC, M (Q_1_, Q_3_)	5.04 (4.44, 5.64)	4.94 (4.36, 5.50)	5.19 (4.56, 5.89)	*Z* = −8.018	<0.001
LDL-C, M (Q_1_, Q_3_)	2.74 (2.23, 3.28)	2.69 (2.20, 3.17)	2.84 (2.33, 3.43)	*Z* = −5.288	<0.001
HDL-C, M (Q_1_, Q_3_	1.51 (1.23, 1.81)	1.54 (1.24, 1.82)	1.46 (1.21, 1.78)	*Z* = −2.841	0.004
TG, M (Q_1_, Q_3_)	1.32 (0.93, 1.92)	1.23 (0.88, 1.74)	1.49 (1.03, 2.16)	*Z* = −8.594	<0.001
FBG, M (Q_1_, Q_3_)	5.30 (4.97, 5.77)	5.17 (4.90, 5.57)	5.58 (5.16, 6.23)	*Z* = −16.018	<0.001
BMI, M (Q_1_, Q_3_)	24.63 (22.59, 26.88)	23.95 (22.10, 26.19)	25.47 (23.40, 27.64)	*Z* = −11.653	<0.001
WC, M (Q_1_, Q_3_)	80.00 (74.00, 88.00)	80.00 (72.00, 86.00)	83.00 (78.00, 90.00)	*Z* = −10.459	<0.001
UA, M (Q_1_, Q_3_)	320.00 (266.00, 377.00)	311.00 (259.00, 366.00)	331.00 (276.00, 390.00)	*Z* = −6.761	<0.001
SBP, M (Q_1_, Q_3_)	132.00 (121.00, 145.67)	123.33 (115.33, 130.33)	149.33 (142.67, 161.00)	*Z* = −48.343	<0.001
DBP, M (Q_1_, Q_3_)	79.67 (72.67, 86.00)	75.00 (69.67, 81.00)	87.33 (81.00, 94.33)	*Z* = −31.845	<0.001
TyG-BMI, M (Q_1_, Q_3_)	31.39 (20.70, 44.84)	28.08 (18.28, 40.14)	36.91 (25.22, 50.32)	*Z* = −13.110	<0.001
Gender, *n* (%)				*χ*^2^ = 13.301	<0.001
Males	1498 (49.97)	851 (56.81)	647 (43.19)		
Females	1500 (50.03)	950 (63.33)	550 (36.67)		
Marriage status, *n* (%)				*χ*^2^ = 13.277	<0.001
Married/cohabiting	2705 (90.23)	1654 (61.15)	1051 (38.85)		
Separated/divorced/widowed/unmarried	293 (9.77)	147 (50.17)	146 (49.83)		
Education level, *n* (%)				*χ*^2^ = 62.065	<0.001
Primary school or below	724 (24.15)	423 (58.43)	301 (41.57)		
Junior middle school	840 (28.02)	421 (50.12)	419 (49.88)		
High school or above	1,434 (47.83)	957 (66.74)	477 (33.26)		
Job, *n* (%)				*χ*^2^ = 147.485	<0.001
Farmers	979 (32.66)	526 (53.73)	453 (46.27)		
Government employees	317 (10.57)	252 (79.50)	65 (20.50)		
Workers	280 (9.34)	168 (60.00)	112 (40.00)		
Sales staff	514 (17.14)	389 (75.68)	125 (24.32)		
others	908 (30.29)	466 (51.32)	442 (48.68)		
Total family income, yuan/*n* (%)				*χ*^2^ = 65.578	<0.001
<20,000	913 (30.45)	454 (49.73)	459 (50.27)		
20,000–59,999	1,087 (36.26)	675 (62.10)	412 (37.90)		
60,000–99,999	524 (17.48)	345 (65.84)	179 (34.16)		
≥1,00,000	474 (15.81)	327 (68.99)	147 (31.01)		
Smoking status, *n* (%)				*χ*^2^ = 3.106	0.078
No	2,404 (80.19)	1,463 (60.86)	941 (39.14)		
Yes	594 (19.81)	338 (56.90)	256 (43.10)		
Drinking status, *n* (%)				*χ*^2^ = 1.548	0.213
No	1,471 (49.07)	867 (58.94)	604 (41.06)		
Yes	1,527 (50.93)	934 (61.17)	593 (38.83)		
PA level, *n* (%)				*χ*^2^ = 59.986	<0.001
Low	435 (14.51)	210 (48.28)	225 (51.72)		
Moderate	479 (15.98)	244 (50.94)	235 (49.06)		
Vigorous	2,084 (69.51)	1,347 (64.64)	737 (35.36)		
DASH score, *n* (%)				*χ*^2^ = 6.022	0.014
<20	867 (28.92)	491 (56.63)	376 (43.37)		
≥20	2,131 (71.08)	1,310 (61.47)	821 (38.53)		
Night sleep duration, *n* (%)				*χ*^2^ = 47.079	<0.001
Insufficient	802 (26.75)	401 (50.00)	401 (50.00)		
Sufficient	1,903 (63.48)	1,220 (64.11)	683 (35.89)		
Excessive	293 (9.77)	180 (61.43)	113 (38.57)		
Diabetes, *n* (%)				*χ*^2^ = 93.897	<0.001
No	2,678 (89.33)	1,689 (63.07)	989 (36.93)		
Yes	320 (10.67)	112 (35.00)	208 (65.00)		
Dyslipidemia, *n* (%)				*χ*^2^ = 61.262	<0.001
No	2,171 (72.41)	1,398 (64.39)	773 (35.61)		
Yes	827 (27.59)	403 (48.73)	424 (51.27)		
Hyperuricemia, *n* (%)				*χ*^2^ = 22.430	<0.001
No	2,600 (86.72)	1,605 (61.73)	995 (38.27)		
Yes	398 (13.28)	196 (49.25)	202 (50.75)		
Overweight/obesity, *n* (%)				*χ*^2^ = 108.818	<0.001
No	1,273 (42.46)	903 (70.93)	370 (29.07)		
	1,725 (57.54)	898 (52.06)	827 (47.94)		
Central Obesity, *n* (%)				*χ*^2^ = 79.832	<0.001
No	2,183 (72.82)	1,418 (64.96)	765 (35.04)		
Yes	815 (27.18)	383 (46.99)	432 (53.01)		

Data are presented as medians (inter quartile range) or number (%). *Z*, Mann–Whitney test; *χ*^2^, Chi-square test; M, Median, Q_1_, 1st Quartile, Q_3_, 3st Quartile; BMI, body mass index; DBP, diastolic blood pressure; FBG, fasting blood glucose; HDL-C, high-density lipoprotein cholesterol; LDL-C, low-density lipoprotein cholesterol; SBP, systolic blood pressure; TC, total cholesterol; TG, triglyceride; TyG-BMI, triglyceride glucose-body mass index; WC, waist circumference; PA, physical activity; DASH, dietary approaches to stop hypertension.

### Baseline characteristics of study participants according to quartiles of TyG-BMI index

Baseline Characteristics of study participants according to quartiles of TyG-BMI index were presented in Table 2. There were significant higher levels of age, TC, LDL-C, HDL-C, TG, FBG, BMI, WC, UA, SBP and DBP in Q4 compared to Q1(all *P* < 0.001). 21.03% of males were in Q1 and 29.44% were in the Q4. 28.93% and 20.60% of males were in the Q1 and Q4. The prevalence of hypertension, diabetes, dyslipidemia, hyperuricemia, overweight/obesity, central obesity were significantly higher in Q4 than Q3 to Q1 (all *P* < 0.001). The proportion of smoking and drinking were significantly higher in Q4 than Q3 to Q1 (all *P* < 0.05). In the bottom quartiles, the level of vigorous physical activity was relatively elevated (*P* < 0.001) (Table 2).

**Table 2:** Baseline characteristics of study participants according to quartiles of triglyceride glucose-body mass index.

Variables	Total (*n* = 2998)	Q1 (*n* = 749)	Q2 (*n* = 750)	Q3 (*n* = 749)	Q4 (*n* = 750)	Statistic value	*P* value
Age, M (Q_1_, Q_3_)	49.00 (42.00, 60.00)	47.00 (39.00, 55.00)	49.00 (42.00, 59.00)	51.00 (43.00, 62.00)	51.00 (44.00, 61.00)	*H* = 63.274	<0.001
TC, M (Q_1_, Q_3_)	5.04 (4.44, 5.64)	4.61 (4.11,5.19)	4.98 (4.41,5.52)	5.09 (4.54,5.73)	5.41 (4.88,6.13)	*H* = 297.353	<0.001
LDL-C, M (Q_1_, Q_3_)	2.74 (2.23, 3.28)	2.41 (1.96, 2.86)	2.74 (2.33, 3.21)	2.89 (2.43, 3.46)	2.95 (2.37, 3.48)	*H* = 206.862	<0.001
HDL-C, M (Q_1_, Q_3_)	1.51 (1.23, 1.81)	1.84 (1.59, 2.12)	1.62 (1.38, 1.87)	1.41 (1.20, 1.60)	1.17 (0.98, 1.38)	*H* = 1049.014	<0.001
TG, M (Q_1_, Q_3_)	1.32 (0.93, 1.92)	0.76 (0.66, 0.87)	1.13 (1.00, 1.30)	1.60 (1.37, 1.85)	2.53 (2.02, 3.40)	*H* = 2417.853	<0.001
FBG, M (Q_1_, Q_3_)	5.30 (4.97, 5.77)	5.04 (4.77, 5.30)	5.19 (4.93, 5.58)	5.43 (5.08, 5.86)	5.88 (5.30, 7.39)	*H* = 661.923	<0.001
BMI, M (Q_1_, Q_3_)	24.63 (22.59, 26.88)	22.31 (20.78, 24.11)	23.80 (22.10, 25.68)	25.35 (23.56, 27.24)	26.95 (25.15, 29.12)	*H* = 916.069	<0.001
WC, M (Q_1_, Q_3_)	80.00 (74.00, 88.00)	76.00 (70.00, 81.00)	80.00 (73.00, 85.00)	82.00 (77.00, 89.00)	86.30 (80.00, 93.00)	*H* = 442.318	<0.001
UA, M (Q_1_, Q_3_)	320.00 (266.00, 377.00)	279.00 (240.00, 327.00)	312.00 (256.25, 363.75)	329.00 (282.00, 384.00)	359.00 (303.00, 422.75)	*H* = 356.885	<0.001
SBP, M (Q_1_, Q_3_)	132.00 (121.00, 145.67)	125.00 (114.33, 136.33)	130.00 (123.67, 146.67)	134.00 (123.67, 146.67)	138.33 (126.33, 150.67)	*H* = 194.462	<0.001
DBP, M (Q_1_, Q_3_)	79.67 (72.67, 86.00)	76.00 (69.00, 82.00)	78.67 (72.33, 85.67)	80.00 (73.33, 86.67)	83.00 (76.33, 90.33)	*H* = 205.833	<0.001
Hypertension, *n* (%)						*χ*^2^ = 163.368	<0.001
No	1801 (60.07)	567 (31.48)	478 (26.54)	425 (23.60)	331 (18.38)		
Yes	1197 (39.93)	182 (15.20)	272 (22.72)	324 (27.07)	419 (35.00)		
Gender, *n* (%)						*χ*^2^ = 42.341	<0.001
Males	1498 (49.97)	315 (21.03)	369 (24.63)	373 (24.90)	441 (29.44)		
Females	1500 (50.03)	434 (28.93)	381 (25.40)	376 (25.07)	309 (20.60)		
Marriage status, *n* (%)						*χ*^2^ = 2.192	0.533
Married/cohabiting	2705 (90.23)	677 (25.03)	676 (24.99)	684 (25.29)	668 (24.70)		
Separated/divorced/widowed/unmarried	293 (9.77)	72 (24.57)	74 (25.26)	65 (22.18)	82 (27.99)		
Education level, *n* (%)						*χ*^2^ = 8.143	0.228
Primary school or below	724 (24.15)	166 (22.93)	197 (27.21)	190 (26.24)	171 (23.62)		
Junior middle school	840 (28.02)	203 (24.17)	198 (23.57)	222 (26.43)	217 (25.83)		
High school or above	1434 (47.83)	380 (26.50)	355 (24.76)	337 (23.50)	362 (25.24)		
Job, *n* (%)						*χ*^2^ = 19.205	0.084
Farmers	979 (32.66)	230 (23.49)	244 (24.92)	240 (24.51)	265 (27.07)		
Government employees	317 (10.57)	94 (29.65)	77 (24.29)	73 (23.03)	73 (23.03)		
Workers	280 (9.34)	84 (30.00)	61 (21.79)	73 (26.07)	62 (22.14)		
Sales staff	514 (17.14)	143 (27.82)	127 (24.71)	122 (23.74)	122 (23.74)		
others	908 (30.29)	198 (21.81)	241 (26.54)	241 (26.54)	228 (25.11)		
Total family income, yuan/*n* (%)						*χ*^2^ = 11.784	0.226
<20,000	913 (30.45)	244 (26.73)	213 (23.33)	221 (24.21)	235 (25.74)		
20,000–59,999	1087 (36.26)	258 (23.74)	293 (26.95)	268 (24.66)	268 (24.66)		
60,000–99,999	524 (17.48)	124 (23.66)	126 (24.05)	153 (29.20)	121 (23.09)		
≥1,00,000	474 (15.81)	123 (25.95)	118 (24.89)	107 (22.57)	126 (26.58)		
Smoking status, *n* (%)						*χ*^2^ = 31.865	<0.001
No	2,404 (80.19)	631 (26.25)	614 (25.54)	609 (25.33)	550 (22.88)		
Yes	594 (19.81)	118 (19.87)	136 (22.90)	140 (23.57)	200 (33.67)		
Drinking status, *n* (%)						*χ*^2^ = 12.084	0.007
No	1,471 (49.07)	394 (26.78)	358 (24.34)	385 (26.17)	334 (22.71)		
Yes	1,527 (50.93)	355 (23.25)	392 (25.67)	364 (23.84)	416 (27.24)		
PA level, *n* (%)						*χ*^2^ = 25.347	<0.001
Low	435 (14.51)	88 (20.23)	125 (28.74)	117 (26.90)	105 (24.14)		
Moderate	479 (15.98)	90 (18.79)	126 (26.30)	139 (29.02)	124 (25.89)		
Vigorous	2,084 (69.51)	571 (27.40)	499 (23.94)	493 (23.66)	521 (25.00)		
Dash score, *n* (%)						*χ*^2^ = 1.983	0.576
<20	867 (28.92)	212 (24.45)	205 (23.64)	223 (25.72)	227 (26.18)		
≥20	2,131 (71.08)	537 (25.20)	545 (25.57)	526 (24.68)	523 (24.54)		
Night sleep duration, *n* (%)						*χ*^2^ = 11.420	0.076
Insufficient	802 (26.75)	183 (22.82)	226 (28.18)	207 (25.81)	186 (23.19)		
Sufficient	1,903 (63.48)	482 (25.33)	452 (23.75)	481 (25.28)	488 (25.64)		
Excessive	293 (9.77)	84 (28.67)	72 (24.57)	61 (20.82)	76 (25.94)		
Diabetes, *n* (%)						*χ*^2^ = 444.230	<0.001
No	2,678 (89.33)	744 (27.78)	728 (27.18)	686 (25.62)	520 (19.42)		
Yes	320 (10.67)	5 (1.56)	22 (6.88)	63 (19.69)	230 (71.88)		
Dyslipidemia, *n* (%)						*χ*^2^ = 1,079.118	<0.001
No	2,171 (72.41)	708 (32.61)	671 (30.91)	589 (27.13)	203 (9.35)		
Yes	827 (27.59)	41 (4.96)	79 (9.55)	160 (19.35)	547 (66.14)		
Hyperuricemia, *n* (%)						*χ*^2^ = 174.840	<0.001
No	2,600 (86.72)	721 (27.73)	682 (26.23)	641 (24.65)	556 (21.38)		
Yes	398 (13.28)	28 (7.04)	68 (17.09)	108 (27.14)	194 (48.74)		
Overweight/obesity, *n* (%)						*χ*^2^ = 658.322	<0.001
No	1,273 (42.46)	555 (43.60)	394 (30.95)	232 (18.22)	92 (7.23)		
Yes	1,725 (57.54)	194 (11.25)	356 (20.64)	517 (29.97)	658 (38.14)		
Central Obesity, *n* (%)						*χ*^2^ = 286.057	<0.001
No	2,183 (72.82)	669 (30.65)	608 (27.85)	508 (23.27)	398 (18.23)		
Yes	815 (27.18)	80 (9.82)	142 (17.42)	241 (29.57)	352 (43.19)		

Data are presented as medians (inter quartile range) or number (%). H, Kruskal-waills test; *χ*^2^, Chi-square test; M, Median, Q_1_, 1st Quartile, Q_3_, 3st Quartile; Q1 to Q4 represents the four quartiles; BMI, body mass index; DBP, diastolic blood pressure; FBG, fasting blood glucose; HDL-c, high-density lipoprotein cholesterol; LDL-C, low-density lipoprotein cholesterol; Q, quartiles; SBP, systolic blood pressure; TC, total cholesterol; TG, triglyceride; TyG-BMI, triglyceride glucose-body mass index; WC, waist circumference; PA, physical activity; DASH, dDietary approaches to stop hypertension.

### The relationship between TyG-BMI index and hypertension by logistic regression analysis

The ORs of TyG-BMI index for hypertension were presented in Table 3. Collinearity diagnostics indicated no multicollinearity among the variables included in the logistic regression model (Supplementary Table 1). It was observed that ORs of hypertension in Q2, Q3, Q4 were 1.773 (95% CI: 1.417–2.217), 2.375 (95% CI: 1.904–2.962), 3.944 (95% CI: 3.163–4.917), respectively in the crude model, compared with Q1. After adjusting for confounding factors, including age, gender, marriage status, education level, job, total family income, smoking status, drinking status, PA level, DASH score, night sleep duration, diabetes, dyslipidemia, hyperuricemia, central obesity, the ORs of hypertension in Q2, Q3, Q4 were 1.641 (1.277–2.109), 1.768 (1.371–2.281), 2.463 (1.794–3.382) in the adjusted model II, respectively. Subgroup analysis in participants with or without diabetes, dyslipidemia or hyperuricemia, central obesity also demonstrated the similar relationship between TyG-BMI index and hypertension (Supplementary Table 2).

**Table 3:** Logistic regression analysis of the relationship between TyG-BMI and hypertension.

TyG-BMI quintiles	Crude model		Adjusted model I		Adjusted model II		Adjusted model III	
	OR (95% CI)	*P* value	OR (95% CI)	*P* value	OR (95% CI)	*P* value	OR (95% CI)	* P* value
Q1	1.000 (Reference)		1.000 (Reference)		1.000 (Reference)		1.000 (Reference)	
Q2	1.773 (1.417–2.217)	<0.001	1.810 (1.406–2.331)	<0.001	1.641 (1.277–2.109)	<0.001	1.653（1.257–2.176）	<0.001
Q3	2.375 (1.904–2.962)	<0.001	2.183 (1.702–2.800)	<0.001	1.768 (1.371–2.281)	<0.001	1.792（1.337–2.402）	<0.001
Q4	3.944 (3.163–4.917)	<0.001	3.895 (3.036–4.998)	<0.001	2.463 (1.794–3.382)	<0.001	2.841（1.683–4.796）	<0.001

Q1 to Q4 represents the four quartiles.

Crude model: unadjusted.

Adjusted model I: adjusted for age, gender, marriage status, education level, job, total family income.

Adjusted model II: adjusted for age, gender, marriage status, education level, job, total family income, smoking status, drinking status, PA level, Dash score, night sleep duration, diabetes, dyslipidemia, hyperuricemia, central Obesity.

Adjusted model III: excluded participants with diabetes, dyslipidemia and hyperuricemia, central obesity, a total of 1,820 participants were included in the sensitivity analysis, adjusted for age, gender, marriage status, education level, job, total family income, smoking status, drinking status, PA level, Dash score, night sleep duration.

PA, physical activity; DASH, dietary approaches to stop hypertension.

### The dose-response relationship between TyG-BMI and hypertension by restricted cubic spline

**Figure 1:**
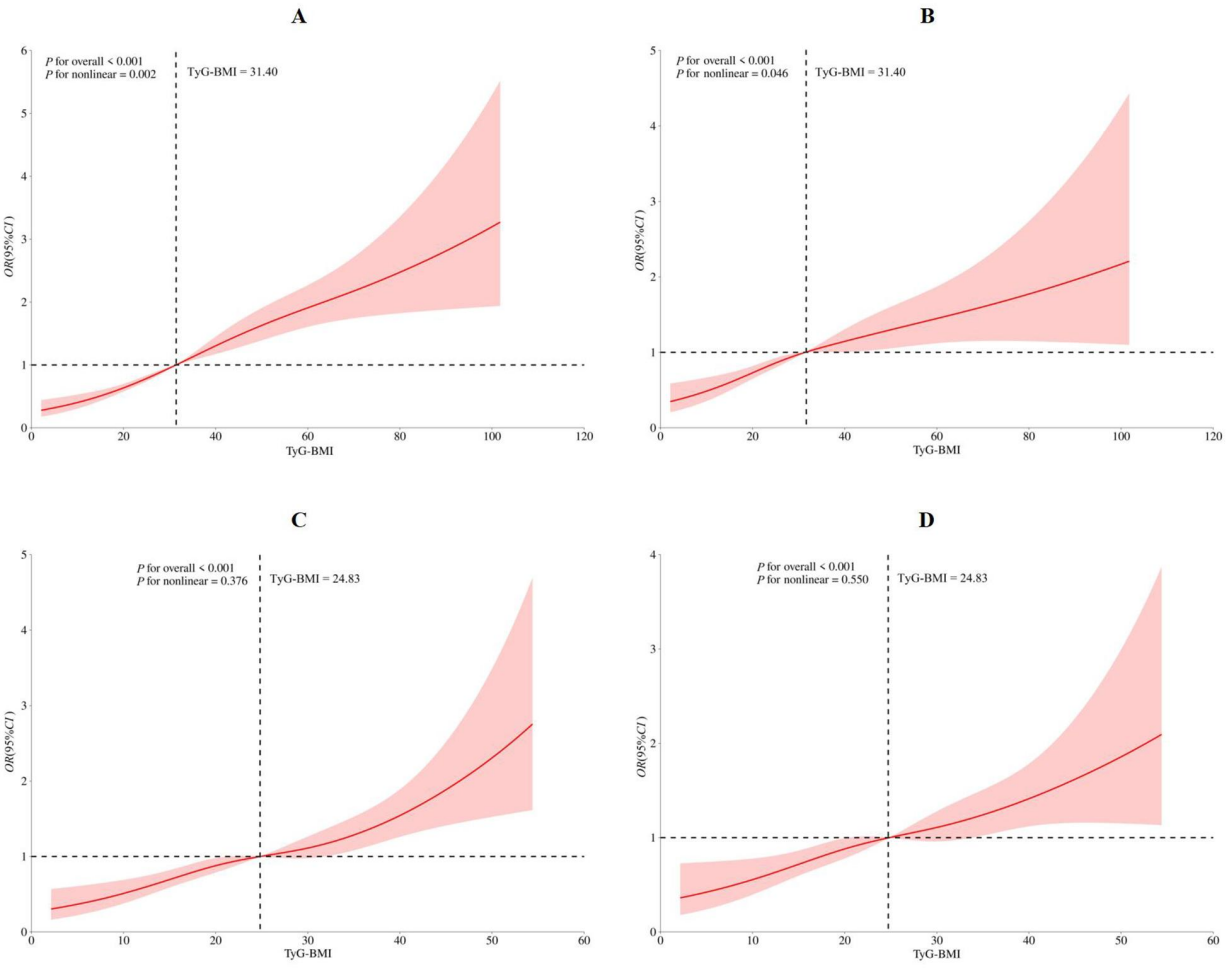
Restricted cubic spine model of the association between triglyceride glucose-body mass index and hypertension **(A)** crude restricted cubic spine model; **(B)** multivanale adiusted restricted cubic spine model with age, gender, marriage status, education level, job, total family income, smoking status, drinking status, PA level, Dash score, night sleep duration, diabetes, dyslipidemia, hyperuricemia, central Obesity; **(C)** sensitivity analysis for crude restricted cubic spine model; **(D)** sensitivity analysis for multivanale adiusted restricted cubic spine model with age, gender, marriage status, education level, job, total family income, smoking status, drinking status, PA level, Dash score, night sleep duration. PA, physical activity; DASH, dietary approaches to stop hypertension.

The RCS model identified four knots located at the 5th, 35th, 65th, and 95th percentiles (Supplementary Table 3). Both the crude (*P*_for overall_ < 0.001, *P*_for nonlinear_ = 0.002, Figure 1A) and the multivariate-adjusted (*P*_for overall_ < 0.001, *P*_for nonlinea*r*_ = 0.046, Figure 1B) RCS models demonstrated a significant nonlinear association between the TyG-BMI index and hypertension risk. The overall upward trend of the curve indicated that higher TyG-BMI levels were associated with increased hypertension risk. When TyG-BMI index was below 31.40, hypertension risk remained relatively low and increased gradually with rising index values. In contrast, when TyG-BMI index exceeded 31.40, hypertension risk was markedly higher and increased more rapidly. Subgroup analysis in participants with or without diabetes, dyslipidemia or hyperuricemia, central obesity also demonstrated the similar relationship between TyG-BMI index and hypertension (Supplementary Figure 1).

### Sensitivity analysis

After excluding participants with diabetes, dyslipidemia, hyperuricemia and central obesity, a total of 1,820 participants were included in the sensitivity analysis, the similar association was demonstrated in the Q2, Q3, and Q4, as 1.653 (1.257–2.176), 1.792 (1.337–2.402), 2.841 (1.683–4.796) in the adjusted model III, respectively (Table 3). Both the crude and multivariate-adjusted RCS models demonstrated the similar relationship between TyG-BMI index and hypertension risk, however, no significant nonlinear relationship was observed in these sensitivity analyses (crude model: *P*_for overall_ < 0.001, *P*_for nonlinea*r*_ = 0.376, Figure 1C; adjusted model: *P*_for overall_ < 0.001, *P*_for nonlinea*r*_ = 0.550, Figure 1D).

### The discriminating ability of the TyG-BMI index by receiver operating characteristic analysis

**Figure 2:**
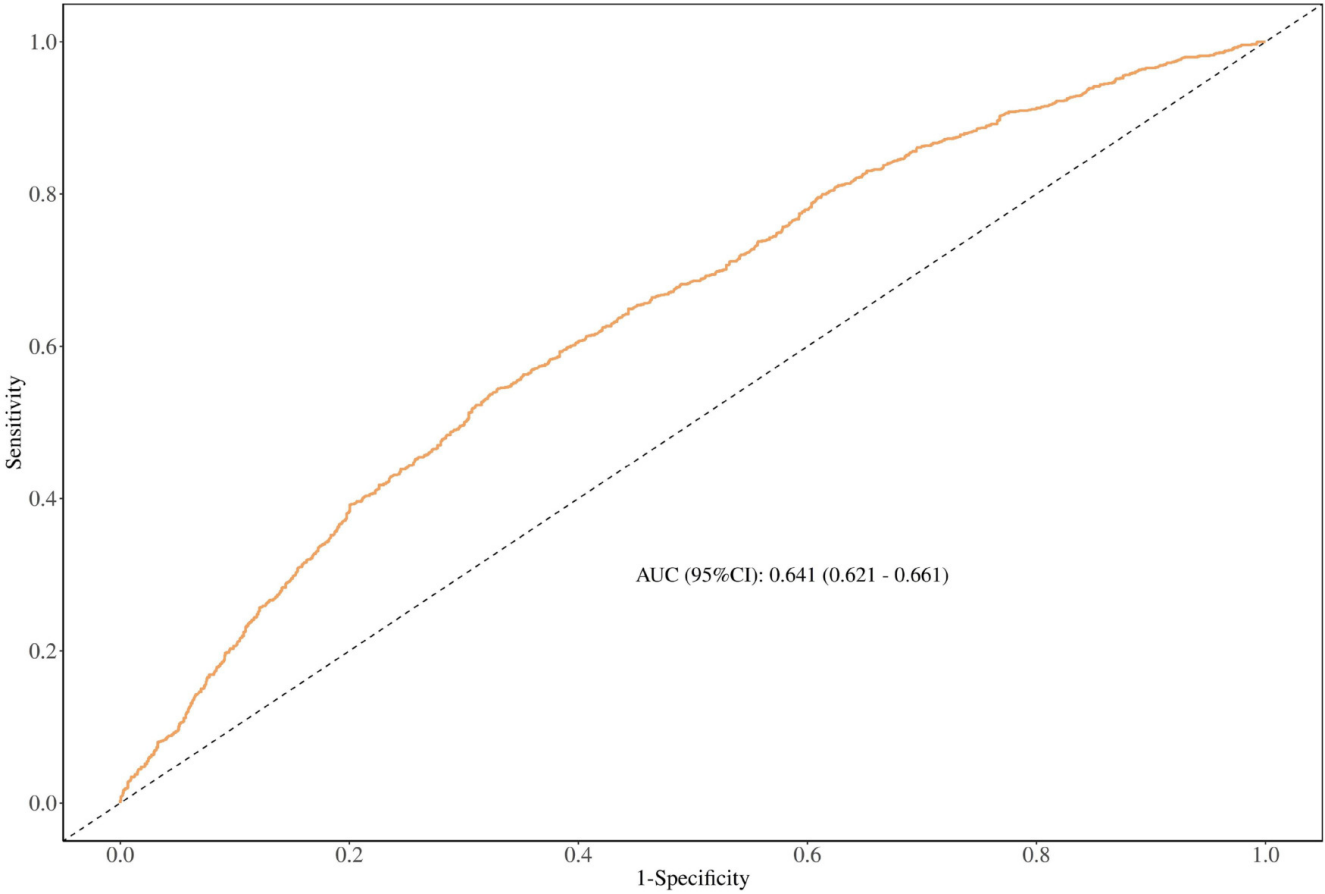
The discriminating ability of the TyG-BMl index by receiver operating characteristic analysis.

The results of ROC analysis for predicting hypertension showed that the TyG-BMI index (AUC: 0.641; 95% *CI*: 0.621, 0.661) had a certain predictive power for hypertension (Figure 2).

## Discussion

The present study investigated the association between the TyG-BMI index and hypertension among rural adults aged 30–79 years in southwest China. Descriptive analysis revealed that TG, FBS, and BMI levels were significantly higher in the highest TyG-BMI quartile (Q4) compared to the lowest (Q1). Our findings indicated that individuals with persistently elevated TyG-BMI levels had a higher prevalence of hypertension. Additionally, participants with high TyG-BMI values were at increased risk of hypertension, even after adjusting for confounding variables using logistic regression when the TyG-BMI index was treated as a categorical variable. The components of the TyG-BMI index: TG, FBS, and BMI were significantly elevated among hypertensive participants compared to normotensive ones. A nonlinear dose–response relationship between TyG-BMI index and hypertension risk was observed using restricted cubic spline analysis when TyG-BMI index was treated as a continuous variable, suggesting a certain predictive capability for hypertension risk. Receiver operating characteristic analysis further confirmed that the TyG-BMI index is a robust predictor of hypertension. Subgroup and sensitivity analyses supported these findings, reinforcing the association between TyG-BMI and hypertension.

Previous studies have found a significant association between the TyG index and hypertension. A cross-sectional study conducted in Korea demonstrated that the TyG-BMI index possessed a strong predictive power for evaluating IR (41), and a cross-sectional study from Japan also demonstrated a strong correlation between the TyG-BMI index and hypertension in individuals with normoglycemia (15). A study from Iran also found that the TyG-BMI index was associated with increased ORs of hypertension (42). A cross-sectional study, involving 92,545 participants showed that the TyG-BMI index was positively related to hypertension (21). Another cross-sectional study involving 60,283 adults in eastern China found that the TyG-BMI index was independently and positively associated with hypertension (6). A retrospective study involving 214,493 participants showed that the TyG-BMI index was independently associated with both pre-hypertension and hypertension in Chinese and Japanese populations (18). Moreover, a prospective nationwide cohort study involving 2,891 participants in China demonstrated a significant positive association between dynamic changes in TyG-BMI and hypertension (43). Another prospective cohort study among middle-aged and older Chinese adults also found a significant positive association between the TyG-BMI index and the subsequent risk of hypertension (44). In addition, ROC analysis demonstrated a certain predictive ability for hypertension, consistent with findings from previous studies (42, 43). Overall, these findings indicate a robust relationship between the TyG-BMI index and hypertension and further underscoring the potential predictive performance for early diagnosis of hypertension by the TyG-BMI index.

The pathophysiological mechanism linking IR and elevated blood pressure via the TyG-BMI index has not been fully elucidated. However, several potential mechanisms have been proposed: (1) Signaling pathways: downregulation of the insulin receptor leads to impaired autophosphorylation and reduced tyrosine phosphorylation of insulin receptor substrates. These changes hinder glucose uptake in adipocytes, skeletal muscle cells, and renal tubules through pathways such as phosphatidylinositol 3-kinase (PI3K). Additionally, inhibition of nitric oxide synthase and activation of the mitogen-activated protein kinase (MAPK) pathway may contribute to vasoconstriction. Together, these alterations may lead to elevated blood pressure (45, 46). (2) Dysfunctional glucose–adipose metabolism: impaired insulin signaling in adipose tissue, along with the accumulation of advanced glycation end products (AGEs), may increase peripheral vascular load by promoting mitochondrial dysfunction and endothelial cell impairment (47, 48). (3) Activation of RAAS and reactive hyperinsulinemia: insulin receptor signaling activates the renin–angiotensin–aldosterone system (RAAS) by promoting the expression of angiotensin II (Ang II) and its receptor, leading to increased plasma volume through enhanced proximal tubular reabsorption of sodium and water. This process contributes to reactive hyperinsulinemia, which further stimulates RAAS activity, thereby forming a vicious cycle (45). (4) Inflammatory response and endothelial cell dysfunction: the inflammatory response and endothelin release occur secondary to RAAS activation, leading to peripheral vasoconstriction (49, 50). (5) Heightened sympathetic nervous system activity: increased sympathetic activity promotes the secretion of epinephrine and norepinephrine, leading to elevated cardiac output and peripheral resistance, along with other mechanisms that contribute to increased blood pressure (51). IR is typically associated with increased levels of circulating insulin, which constitutes a certain cause of hypertension (45) because it determines vasoconstiction by increasing endothelin-1 levels, at the expense of a reduction in NO synthesis, and increasing circulating norepinephrine (46, 51). Therefore, IR measurements prove to be valuable monitoring tools the progression of hypertension.

Currently, there is a lack of tools to predict hypertension, making it difficult for the general public to know their risk of developing hypertension and to manage it effectively (15). Although the HECT is currently considered the gold standard for detecting IR, its application in large-scale epidemiological studies is limited due to complexity and cost. Therefore, indirect IR indices can serve as practical alternatives in such settings. However, HOMA-IR remains difficult to implement in economically underdeveloped regions (52). The TyG-BMI index, a recently proposed metric combining BMI and TyG parameters, is increasingly recognized as a reliable indicator for assessing IR in clinical practice (43). Previous studies in Guangdong (21), eastern China (6) and the Yangtze River Delta of China (22) had suggested a positive association between TyG-BMI index and hypertension in Chinese population. However, the association between the TyG-BMI index and hypertension has not been fully validated in rural southwest China. Based on previous studies, the TyG-BMI index may serve as a cost-effective tool for assessing hypertension risk. As it requires minimal laboratory procedures and can be easily obtained from a single participant, the TyG-BMI index is more feasible for use in epidemiological studies and clinical practice compared to the HIEC and HOMA-IR (43). Moreover, the TyG-BMI index may provide a more holistic assessment of hypertension risk, which is superior to TyG index alone (24). In contrast, the TyG-BMI index is not only easy to calculate from routinely available data but is also unaffected by regional development levels, making it suitable for both developed and underdeveloped settings.

According to Liu's 2017 study, the prevalence of hypertension in the southwest Chinese population was 23.90%, with rural residents exhibiting a higher prevalence than their urban counterparts (26.0% vs. 21.6%) (53). Nationally, the prevalence of hypertension among adults in China was 31.60% (4), and the prevalence of hypertension in rural areas was 33.70%, which was higher than the urban areas (29.10%). Globally, between 1990 and 2019, the number of adults aged 30–79 years with hypertension increased from 650 million to 1.28 billion, nearly half of whom were unaware of their condition (54). Therefore, early identification of individuals at risk for hypertension facilitates effective blood pressure management and the prevention of hypertension-related cardiovascular and cerebrovascular diseases, particularly in economically underdeveloped regions.

The TyG-BMI index contributes to the primary prevention of hypertension by incorporating indicators related to weight management and metabolic risk control, which can be influenced through regular physical activity and a healthy diet. By integrating both insulin resistance and body mass index, the TyG-BMI index offers a more comprehensive assessment of an individual's metabolic status. Measuring fasting blood glucose, triglycerides, and BMI enables the early identification of individuals at high risk for hypertension, even before typical symptoms appear. Such early detection is crucial, as hypertension can cause cardiovascular damage long before it becomes clinically evident. An elevated TyG-BMI index is often associated with unhealthy lifestyle factors, including high-calorie diets, physical inactivity, smoking, and excessive alcohol consumption (55–57). By monitoring the TyG-BMI index, personalized health recommendations can be provided, such as modifying dietary habits, increasing physical activity, quitting smoking, and limiting alcohol consumption, to reduce the risk of hypertension. For individuals already diagnosed with hypertension, the TyG-BMI index can assist clinicians in assessing metabolic status and developing more effective treatment strategies. For instance, in patients with elevated TyG-BMI, in addition to conventional antihypertensive therapy, medications that enhance insulin sensitivity, such as metformin, may be considered for the integrated management of blood pressure and metabolic abnormalities. Early screening and intervention using the TyG-BMI index may help lower the incidence of hypertension and its complications, thereby alleviating the burden on healthcare systems. Preventing and treating hypertension at an early stage can avert serious complications such as cardiovascular and kidney diseases, which are often expensive to manage. Moreover, the widespread use of the TyG-BMI index can enhance public awareness of hypertension and its risk factors, promote healthier lifestyles, and ultimately reduce the societal prevalence of hypertension while improving overall population health.

We believe that this study is the first to investigate the association between the TyG-BMI index and hypertension among adults in rural southwest China. The results indicate that the TyG-BMI index is significantly associated with an increased risk of hypertension, whether considered as a continuous or categorical variable. Additionally, both subgroup and sensitivity analyses were performed to further confirm the robustness of this association. Due to the rigorous statistical methods applied, the findings of this study are considered highly reliable. However, several limitations should be acknowledged. First, the cross-sectional design limits the ability to infer causality. Second, the study population was restricted to adults aged 30–79 years in rural southwest China, which may limit the generalizability of the findings to other populations. Third, some participants were aware of their hypertension status and may have adopted interventions to lower their blood pressure, potentially introducing Neyman bias. This type of bias may lead to an underestimation or misestimation of the association between the TyG-BMI index and hypertension. Lastly, we were unable to compare the TyG-BMI index with HIEC, the gold standard for diagnosing insulin resistance, due to the constraints of the population-based cross-sectional design.

On June 6, 2024, 16 government departments, including the National Health Commission, jointly issued the Implementation Plan for the “Year of Weight Management” Activities. The plan aims to promote effective weight management over a three-year period beginning in 2024. By the end of this period, the supportive environment for weight management, public awareness, and individual skills related to weight control, as well as adherence to healthy lifestyles, are expected to improve significantly. Additionally, abnormal weight conditions among certain high-risk populations are anticipated to be mitigated. The TyG-BMI index, as an emerging indicator, is expected to play an important role in supporting the objectives of the “Year of Weight Management.”

## Conclusion

Overall, the present study demonstrated that the TyG-BMI index is significantly associated with an increased risk of hypertension. These findings support the hypothesis that the TyG-BMI index may serve as a novel predictive marker for the early identification of individuals at risk for hypertension. By optimizing BMI, the risk of developing hypertension may be reduced. Furthermore, the TyG-BMI index holds promise as a surveillance tool for future public health education and primary prevention initiatives targeting hypertension. Lifestyle modifications, such as weight management, regular physical activity, and adherence to a healthy diet—can improve insulin resistance and contribute to hypertension prevention.

## Data Availability

The original contributions presented in the study are included in the article/Supplementary Material, further inquiries can be directed to the corresponding author.
