# Supplementary material for: The triglyceride glucose-body mass index is positively associated with higher risk of hypertension in rural southwest Chinese population: a cross sectional study
Source: Front Cardiovasc Med. 2026 Feb 2;12:1677048. doi: 10.3389/fcvm.2025.1677048 (PMC12907333; doi:10.3389/fcvm.2025.1677048)
Supplement: Supplementary file 1 [file Table1.doc]

Supplementary table 1. Multicollinearity diagnostics for variables included in the logistic regression analysis

| Variables | Tolerance | VIF |
| --- | --- | --- |
| TyG-BMI | 0.504 | 1.985 |
| Age | 0.761 | 1.314 |
| Gender | 0.671 | 1.490 |
| Marriage status | 0.965 | 1.036 |
| Education level | 0.961 | 1.041 |
| Job | 0.902 | 1.109 |
| Total family income, | 0.902 | 1.109 |
| Smoking status | 0.739 | 1.353 |
| Drinking status | 0.885 | 1.130 |
| PA level | 0.823 | 1.215 |
| DASH score | 0.971 | 1.030 |
| Night sleep duration | 0.959 | 1.043 |
| Diabetes | 0.840 | 1.190 |
| Dyslipidemia | 0.698 | 1.433 |
| Hyperuricemia | 0.855 | 1.170 |
| Overweight/obesity | 0.702 | 1.424 |
| Central Obesity | 0.810 | 1.235 |

VIF. variance inflation factor;

TyG-BMI. triglyceride glucose-body mass index;

PA. physical activity;

DASH. dietary approaches to stop hypertension.
