# Supplementary material for: The triglyceride glucose-body mass index is positively associated with higher risk of hypertension in rural southwest Chinese population: a cross sectional study
Source: Front Cardiovasc Med. 2026 Feb 2;12:1677048. doi: 10.3389/fcvm.2025.1677048 (PMC12907333; doi:10.3389/fcvm.2025.1677048)
Supplement: Supplementary file 2 [file Table2.doc]

Supplementary Table 2. Logistic regression analysis of the relationship between TyG-BMI and hypertension in participants with or without diabetes, dyslipidemia, hyperuricemia or central obesity .

| Variables | No | | |  | Yes | | |
| --- | --- | --- | --- | --- | --- | --- | --- |
|  | *OR* | 95%CI | *P* value |  | *OR* | 95%CI | *P* value |
| Diabetes^a^ |  |  |  |  |  |  |  |
| Q1 | 1.000 |  |  |  | 1.000 |  |  |
| Q2 | 1.597 | 1.232-2.070 | ＜0.001 |  | 1.389 | 0.114-16.894 | 0.797 |
| Q3 | 1.632 | 1.241-2.146 | ＜0.001 |  | 1.682 | 0.161-17.583 | 0.664 |
| Q4 | 2.519 | 1.766-3.592 | ＜0.001 |  | 0.809 | 0.078-8.428 | 0.859 |
| Dyslipidemia^b^ |  |  |  |  |  |  |  |
| Q1 | 1.000 |  |  |  | 1.000 |  |  |
| Q2 | 1.605 | 1.225-2.104 | 0.001 |  | 1.416 | 0.587-3.416 | 0.439 |
| Q3 | 1.733 | 1.297-2.316 | ＜0.001 |  | 1.308 | 0.580-2.952 | 0.517 |
| Q4 | 1.874 | 1.221-2.878 | 0.004 |  | 2.134 | 0.959-4.745 | 0.063 |
| Hyperuricemia^c^ |  |  |  |  |  |  |  |
| Q1 | 1.000 |  |  |  | 1.000 |  |  |
| Q2 | 1.640 | 1.268-2.123 | ＜0.001 |  | 2.397 | 0.734-7.832 | 0.148 |
| Q3 | 1.715 | 1.316-2.235 | ＜0.001 |  | 3.838 | 1.216-12.109 | 0.022 |
| Q4 | 2.281 | 1.625-3.201 | ＜0.001 |  | 6.404 | 1.91-21.472 | 0.003 |
| Central obesity^d^ |  |  |  |  |  |  |  |
| Q1 |  |  |  |  |  |  |  |
| Q2 | 1.699 | 1.290-2.237 | ＜0.001 |  | 1.580 | 0.828-3.015 | 0.165 |
| Q3 | 1.539 | 1.149-2.061 | 0.004 |  | 2.888 | 1.578-5.289 | 0.001 |
| Q4 | 2.061 | 1.401-3.033 | ＜0.001 |  | 3.972 | 2.065-7.639 | ＜0.001 |

TyG-BMI. triglyceride glucose-body mass index; OR. Odds ratio; CI. confidence interval;

Q1, Q2, Q3, Q4: quartiles for TyG-BMI

a. adjusted for age, gender, marriage status, education level, job, total family income, smoking status, drinking status, PA level, DASH score, night sleep duration, dyslipidemia, hyperuricemia, central obesity;

b. adjusted for age, gender, marriage status, education level, job, total family income, smoking status, drinking status, PA level, DASH score, night sleep duration, diabetes, hyperuricemia, central obesity;

c. adjusted for age, gender, marriage status, education level, job, total family income, smoking status, drinking status, PA level, DASH score, night sleep duration, diabetes, dyslipidemia, central obesity;

d. adjusted for age, gender, marriage status, education level, job, total family income, smoking status, drinking status, PA level, DASH score, night sleep duration, diabetes, dyslipidemia, hyperuricemia.

PA. physical activity; DASH. dietary approaches to stop hypertension.
