# Supplementary material for: The triglyceride glucose-body mass index is positively associated with higher risk of hypertension in rural southwest Chinese population: a cross sectional study
Source: Front Cardiovasc Med. 2026 Feb 2;12:1677048. doi: 10.3389/fcvm.2025.1677048 (PMC12907333; doi:10.3389/fcvm.2025.1677048)
Supplement: Supplementary file 3 [file Table3.doc]

Supplementary table 3. TyG-BMI values corresponding to the RCS knots

| Model | Knot 1 | Knot 2 | medium | Knot 3 | Knot 4 |
| --- | --- | --- | --- | --- | --- |
| A | 10.12 | 25.08 | 31.40 | 38.70 | 67.78 |
| B | 10.12 | 25.08 | 31.40 | 38.70 | 67.78 |
| C | 8.19 | 20.02 | 24.83 | 29.70 | 44.15 |
| D | 8.19 | 20.02 | 24.83 | 29.70 | 44.15 |

1. crude restricted cubic spine model;
2. multivanale adiusted restricted cubic spine model with age, gender, marriage status, education level, job, total family income, smoking status, drinking status, PA level, Dash score, night sleep duration, diabetes, dyslipidemia, hyperuricemia, central Obesity;
3. sensitivity analysis for crude restricted cubic spine model;
4. sensitivity analysis for multivanale adjusted restricted cubic spine model with age, gender, marriage status, education level, job, total family income, smoking status, drinking status, PA level, Dash score, night sleep duration. PA. Physical activity; DASH. Dietary approaches to stop hypertension.

The first knot was placed at the 5th percentile, the second knot at the 35th percentile, the third knot at the 65th percentile, and the fourth knot at the 95th percentile.
