# Supplementary figures and images for: The triglyceride glucose-body mass index is positively associated with higher risk of hypertension in rural southwest Chinese population: a cross sectional study
Source: Front Cardiovasc Med. 2026 Feb 2;12:1677048. doi: 10.3389/fcvm.2025.1677048 (PMC12907333; doi:10.3389/fcvm.2025.1677048)

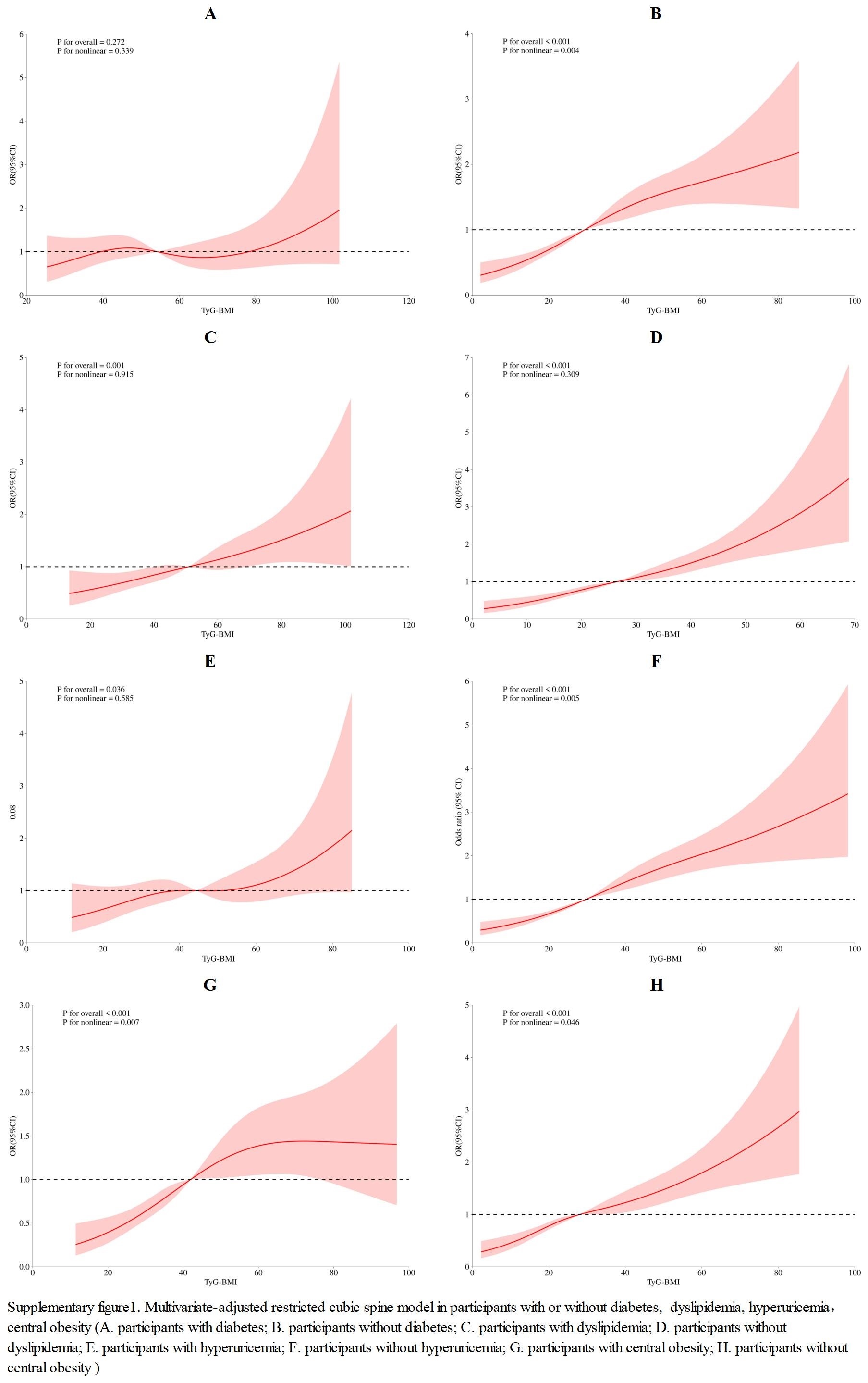

Supplement: Supplementary file 5 [file Image1.jpeg]
